# Quality of antenatal care in rural Tanzania: counselling on pregnancy danger signs

**DOI:** 10.1186/1471-2393-10-35

**Published:** 2010-07-01

**Authors:** Andrea B Pembe, Anders Carlstedt, David P Urassa, Gunilla Lindmark, Lennarth Nyström, Elisabeth Darj

**Affiliations:** 1 Department of Obstetrics and Gynaecology, Muhimbili University of Health and Allied Sciences, Dar es Salaam, Tanzania; 2 Department of Women's and Children's Health, International Maternal and Child Health (IMCH), Uppsala University, Uppsala, Sweden; 3 Department of Surgery, Central Hospital, Karlstad, Sweden; 4 Department of Community Health, Muhimbili University of Health and Allied Sciences, Dar es Salaam, Tanzania; 5 Department of Public Health and Clinical Medicine, Umeå University, Umeå, Sweden

## Abstract

**Background:**

The high rate of antenatal care attendance in sub-Saharan Africa, should facilitate provision of information on signs of potential pregnancy complications. The aim of this study was to assess quality of antenatal care with respect to providers' counselling of pregnancy danger signs in Rufiji district, Tanzania.

**Methods:**

A cross-sectional study was conducted in 18 primary health facilities. Thirty two providers were observed providing antenatal care to 438 pregnant women. Information on counselling on pregnancy danger signs was collected by an observer. Exit interviews were conducted to 435 women.

**Results:**

One hundred and eighty five (42%) clients were not informed of any pregnancy danger signs. The most common pregnancy danger sign informed on was vaginal bleeding 50% followed by severe headache/blurred vision 45%. Nurse auxiliaries were three times more likely to inform a client of a danger sign than registered/enrolled nurses (OR = 3.7; 95% CI: 2.1-6.5) and Maternal Child Health Aides (OR = 2.3: 95% CI: 1.3-4.3) and public health nurses (OR = 2.5; CI: 1.4-4.2) were two times more likely to provide information on danger signs than registered/enrolled nurses. The clients recalled less than half of the pregnancy danger signs they had been informed during the interaction.

**Conclusion:**

Two out of five clients were not counselled on pregnancy danger signs. The higher trained cadre, registered/enrolled nurses were not informing majority of clients pregnancy danger signs compared to the lower cadres. Supportive supervision should be made to enhance counselling of pregnancy danger signs. Nurse auxiliaries should be encouraged and given chance for further training and upgrading to improve their performance and increase human resource for health.

## Background

It was estimated in 2005 that of more than 500 000 maternal deaths worldwide, more than half occurred in sub-Saharan Africa [1]. The same source estimates lifetime risk of maternal death to be 1 in 16 in sub-Saharan Africa, as compared to 1 in 2800 in developed regions. A majority of these maternal deaths are considered preventable, as are newborn deaths, if there is timely access to appropriate interventions when obstetric complications occur. Of note, three-quarters of the 4 million global neonatal deaths occur in the first week of life [2], and the stillbirth rate is 32 stillbirths per 1000 deliveries, of which 24 - 37% occur during the intrapartum period [3]. Confronting these estimates is the supposition that health education and information conveyed to mothers about potential risks, as well as the counselling of danger signs, might lead to improved early decision making and care-seeking where access to health facilities is concerned.

The most recent estimates coming from Tanzania show that 94% of pregnant women make at least one antenatal care visit, while only 62% make four or more visits [4]. Women are supposed to be educated and counselled regarding pregnancy-related danger signs during these visits, and that a delivery plan will be created so that readiness for emergency can be better assured. Counselling on pregnancy danger signs is to be conducted according to focused antenatal care (FANC) guidelines, which include signs such as vaginal bleeding, severe headache or blurred vision, severe abdominal pain, swollen hands and face, fever, baby stopped or reduced movement, and excessive tiredness/breathlessness [5]. However, studies conducted in Tanzania report low awareness of these danger signs among women with delivery experience [6,7]. Moreover, only 47% of women attending antenatal visits recall having been informed of such complications [4]. The Tanzanian situation is not unlike that found in other sub-Saharan countries. Nikiema and co-workers [8] utilized data from the Demographic and Health Survey and found that in 15 of 19 sub-Saharan countries, less than half of all women recalled having received information during their antenatal care about the potential danger signs of pregnancy complications.

We focus here on the quality of antenatal care in Rufiji rural district, Tanzania, with respect to provider counselling of pregnancy danger signs.

## Methods

### Study area

Rufiji district has been described in detail elsewhere [6,9], however, it suffices to describe here that study area is located about 200 km south of the capital, Dar es Salaam, and has a population estimated at 240 000 people [10]. There are two hospitals in the district. One facility is a government district hospital while the other is owned by a religious organization. Both hospitals provide antenatal care, as well as comprehensive emergency obstetric care for the entire region. Referrals are made for high risk pregnancy or complications from 52 dispensaries and 4 rural health centres (RHCs) found within the district, which also provide antenatal care, as stipulated by the national guidelines on the Reproductive and Child Health Card Number 4 (RCHC-4) [11].

The dispensaries and RHCs are staffed by prescribers and nurses. Prescribers are comprised mainly of Assistant Clinical Officers and Clinical Officers, who are consulted when there is need for treatment or if referral is indicated. Nurses represent the main providers of antenatal and delivery care in all facilities within the district, and these comprise registered as well as enrolled nurses, who have been trained at various levels of the health system for at least four years in nursing and midwifery care. The earlier "public health nurse" cadre has been recently phased out, but these providers were trained for three years to provide maternal and child care services as a public health service. Maternal and Child Health Aides (MCHAs) have two years training as auxiliary midwives, and provide antenatal and postnatal care, family planning services, counselling and treatment of sexually transmitted infections, but also attend normal deliveries commonly occurring at the dispensaries and health centres. Auxiliary nurses have one-year training in nursing and/or on-the-job training. These are regarded as not trained to provide skilled care, but due to a shortage of trained staff, they are providing maternal care in some of the dispensaries.

The number of women attending antenatal care depends on the catchment population of the health facility. Antenatal care is provided in the district during weekdays from 7:30 am to 3:30 pm, and in most of maternal health facilities, public education is available as a lecture presentation given by providers before opening hours of the antenatal clinic (topic choice is decided by the provider). Further, all RHCs and some dispensaries provide outreach services once a week. These services include vaccination for pregnant mothers and children, growth monitoring for under-fives, and health education.

### Study design and data collection

A cross-sectional study was performed between March and July 2008 of women attending antenatal care as well as all providers in select health facilities. The study population was stratified by type of health institution the women visited for antenatal care. A stratified random sample of health institutions was used to minimize potential variance resulting from strata having only a few subjects. The sampling fraction among the four RHCs was 100%, and 29% for the 14 dispensaries involved in a follow-up study of referred women [*Pembe et al, unpublished results*]. Our random sample therefore fulfils the recommendation for incorporating at least 25% of the health facilities within the district, thereby making an adequate representation of a district health service situation [12,13]. We assumed that 50% of the women could have been counselled on pregnancy danger signs, with an estimated precision of 5% and the 95% confidence interval and a refusal rate of 10%. A sample of 427 women was thus calculated. All clients attending antenatal care on the day of the data collection were included. Each health facility was visited twice for data collection in order to achieve the calculated sample.

Data collection involved observation of the client-provider interaction session, as well as exit interviews with the clients following the session. Two experienced registered nurses acted as research assistants, and were trained in conducting the observations and exit interviews. They received further training on the identification of high risk pregnancies, according to the RCHC-4, and potential pregnancy danger signs, birth preparedness and complication readiness according to the FANC manual.

The first author and the two research assistants visited each health facility over a period of two days per facility. On the first day, introductions were made to the providers, and an explanation was made of the study aims. The team was also able during this initial visit to become familiar with the surroundings and to collect facility information. An emphasis was made that the researchers were not representing the Ministry of Health and Social Welfare, and that the findings would by no means be used against the staff members. The second day was used to collect data. Clients were informed of the objective of the study while in the consultation room, and permission was obtained at that time for the research assistant to be present in the room to observe the client-provider interaction. All clients who were approached agreed to the request and participated.

#### Client-provider interaction

While in the consultation room activities included history-taking, examination, health education and counselling, as well as documentation of the findings. A pre-tested, structured observation checklist was used to record consultation interaction between the clients and providers. The checklist of collected variables included counselling for pregnancy danger signs, identification of high risk pregnancies, provision of advice for referral and the place of delivery. Interaction time, the time the client spent with the provider, was recorded using a stopwatch.

#### Exit interview

After the observation, clients were given a card with an identification number to forward to the research assistant outside the consultation room. The exit interviews were conducted at a convenient place within the health facility premises, and away from providers and other women attending the antenatal clinic. A pre-tested, structured questionnaire was used, which contained questions on basic demographic information, the number of visits to the health care facility, duration of the pregnancy, gravidity and parity. Demographic information given was validated against the RCHC-4 card. Clients were further asked whether they were informed during the visit to go to the health facility if any danger signs related to the pregnancy were noticed.

### Data analysis

Data were computerised using Epi Info and analysed using SPSS version 14. Median test and Mann-Whitney U test were used to compare median durations of interaction between the four categories of providers and between registered/enrolled nurses and other cadres, respectively. The differences were deemed significant when p < 0.05. Bivariate logistic regression analysis was used to identify factors associated with clients who had been counselled on pregnancy danger signs. Independent variables significant in the bivariate analysis were then entered into a multivariate logistic regression analysis. The associations were estimated by odds ratio (OR) and 95% confidence interval (CI).

### Ethical consideration

Approval for the study was granted by the Senate Research and Publication Committee of Muhimbili University of Health and Allied Sciences, Tanzania. Permission to conduct the study was obtained from Rufiji District authorities, and the charge authorities for each of the health facilities. Written informed consent was obtained from all clients and providers who participated in the study. In cases where the research assistants identified an obstetric danger sign or a reason for referral that was missed by the provider, such information was communicated back to the provider as well as the client immediately following the exit interview and before the client left the health facility.

## Results

Of the total dispensaries, there were six that had only nurse auxiliaries to provide maternal care services, while two dispensaries were operated only by MCHAs. In the remaining of the facilities the services were provided by the public health nurses and/or registered/enrolled nurses with assistance from the nurse auxiliaries and MCHAs. Antenatal care was given by 32 providers to a total of 438 clients. However, three clients dropped out of the study after the observation session, leaving 435 for exit interviews. Observations result from 11 auxiliary nurse cadres who conducted 118 client interactions, 5 MCHAs who conducted 87 interactions, 9 public health nurses who had 128 interactions, and 7 registered/enrolled nurses who had 102 interactions.

Median age of the clients was 27 years (Range: 14-46 years), and 84% were married/cohabiting, 37% had no formal education, 49% had completed primary education, and 77% were socially marginalized as peasants. One-fifth of the clients were primigravidae, 34% were booking for antenatal care (first visit), and 49% were in their last trimester of the pregnancy.

**Figure 1:**
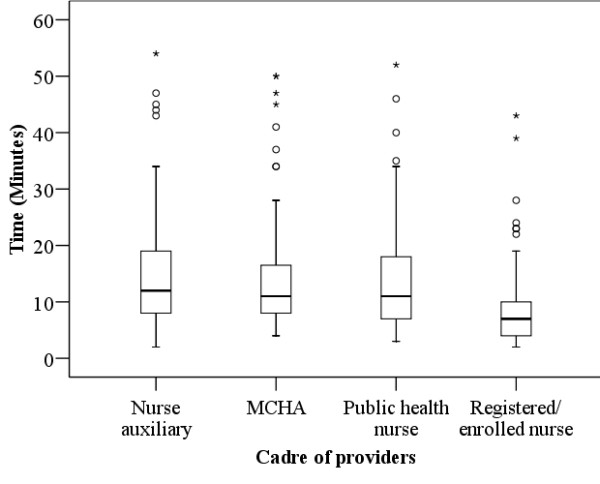
**Box plot diagram of client-provider interaction time by cadre of providers**.

The median number of observed interactions per provider was 14 (Range: 2-61), and median client-provider interaction time was 10 minutes (Range: 2-54 minutes). Clients on their first visit for antenatal care had a mean interaction time with provider of 17 minutes while those revisit had 10 minutes (p < 0.001). As shown in Figure 1, the registered/enrolled nurses had less interaction time with clients compared to other cadres (p < 0.001). The communication between the client and provider was primarily unidirectional, with the provider giving the information and the clients listening. Only half of the clients were provided a chance to ask questions. In 76 of the 435 (18%) interactions, clients were not informed of the progress of their pregnancies.

**Figure 2:**
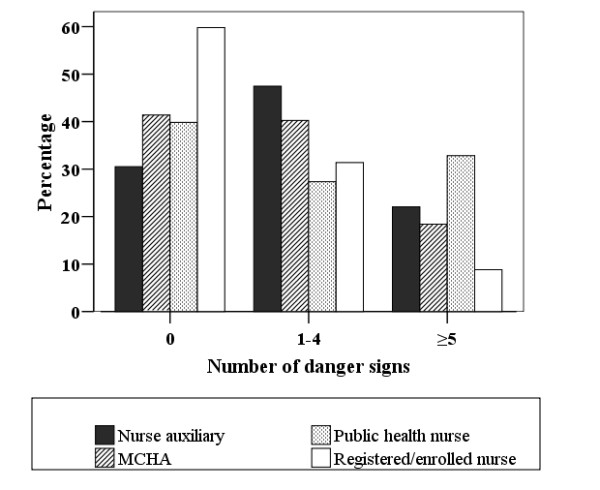
Percent of clients who informed of 0, 1-4 or ≥5 pregnancy danger signs, according to the cadre of provider

Two out of five (42%) clients were not informed of any pregnancy danger sign. Only 8.7% clients were informed of all seven danger signs. The median number of pregnancy danger signs informed was 2 (Range: 0-7). Figure 2 shows the distribution of clients informed of danger signs per cadre. Of note, 60% of clients attended by registered/enrolled nurses were not informed of any danger signs, as compared to nurse auxiliaries 30%, MCHA 41% and public health nurse 40%.

Table 1 presents the danger signs most commonly informed by the providers, which were vaginal bleeding 50% and severe headache/blurred vision 45%, while the least informed danger sign was excessive tiredness or breathlessness. On exit interview, women were able to recall less than half of the informed pregnancy danger signs. Higher recall was observed for vaginal bleeding and severe headache or blurred vision.

**Table 1:** Clients informed by providers and recalled on exit interviews pregnancy danger signs (n = 435).

Pregnancy danger sign	Informed		Recalled	
		
	n	%	n	%
Vaginal bleeding	219	50	162	74
Severe headache or blurred vision	197	45	109	55
Severe abdominal pain	143	33	55	38
Swollen hands and face	140	32	64	46
Fever	120	28	34	28
Baby stops or reduce moving	114	26	17	15
Excessive tiredness/breathlessness	83	19	15	18

The independent variables; age of the mother, marital status, educational level, gravidity and having been told of a need for referral by the provider were not associated with being counselled on pregnancy danger signs in the bivariate logistic regression analysis. The remaining independent variables of occupation, number of antenatal care visits, gestational age at the time of antenatal visit, as well as type of provider cadre, were included in the multivariate logistic regression analysis. Nurse auxiliaries were more than three times likely to inform a client of a pregnancy danger sign than registered/enrolled nurses (OR = 3.7; 95% CI: 2.1-6.5), while MCHAs and public health nurses were two times more likely to inform a client pregnancy danger sign than registered/enrolled nurses (OR = 2.3; 95% CI 1.3-4.3) and (OR = 2.5; 95% CI: 1.4-4.2), respectively (Table 2).

**Table 2:** Bivariate and multivariate logistic regression analysis of the likelihood to be informed of at least one danger sign.

Characteristic	Counselled on danger sign		Bivariate analysis		Multivariate analysis	
			
	Yes	No	OR	95% CI	OR	95% CI
*Age*						
≤19	40	21	1.4	0.77-2.7		
20-29	130	102	0.96	0.63-1.5		
≥30	81	61	1			
*Marital status*						
Single	45	24	1.5	0.85-2.5		
Married/cohabiting	206	160	1			
*Educational level*						
No formal education	101	59	1.4	0.96-2.1		
Primary education or above	150	125	1			
*Occupation*						
Peasant	203	132	1.7	1.1-2.6	1.5	0.96-2.5
Other	48	52	1		1	
*Gravidity*						
Primigravida	56	33	1.3	0.81-2.1		
Multigravida	195	151	1			
*Number of antenatal visits*						
First visit	96	51	1.6	1.1-2.4	1.5	0.94-2.5
Revisit	155	133	1		1	
*Gestational age (weeks)*						
≤27	141	83	1.6	1.1-2.3	1.4	0.86-2.1
≥28	110	101	1		1	
*Indication for referral*						
Yes	101	59	1.4	0.96-2.1		
No	150	125	1			
*Cadre of provider*						
Nurse auxiliary	82	36	3.4	1.9-5.9	3.7	2.1-6.5
MCHA	51	36	2.1	1.2-3.8	2.3	1.3-4.3
Public health nurse	77	51	2.2	1.3-3.8	2.5	1.4-4.2
Registered/enrolled nurse	41	61	1		1	

Odds ratio (OR) and 95% confidence intervals (CI) (n = 435)

## Discussion

Despite efforts made by the Tanzanian government to improve quality of antenatal care, this study demonstrated deficiencies in the counselling of pregnancy danger signs in this rural district. A significant proportion of the clients were not informed of pregnancy danger signs, and the interaction time between clients and providers was of too short duration. The highest cadre (registered/enrolled nurses) was least likely to inform about pregnancy danger signs than the lower cadres (nurse auxiliaries MCHAs, public nurses), and had the least interaction time with clients.

Informing antenatal clients on pregnancy danger signs, and developing an emergency contingency plan, to women living in rural areas is important for decreasing geographical and financial barriers [8,14]. Client awareness of potential pregnancy danger signs is expected to decrease the amount of time between when a complication occurs, to the time when the complication is identified and the decision is made to seek emergency obstetric care. Providers in our study generally performed poorly in informing the clients on pregnancy danger signs. Similar results are documented for other parts of Tanzania and sub-Saharan countries [4,8,15,16]. Further, poor counselling of the pregnancy danger signs is considered to be one of the reasons for poor awareness of danger signs among women who have attended antenatal care [6,7,17,18]. This means there is a need to ensure that providers inform all antenatal care clients about the pregnancy danger signs. Strengthening supportive supervision from the District Health Management Team in order to improve counselling during antenatal care is therefore crucial.

Despite the nurse auxiliaries being regarded as the unskilled cadre, they performed better in informing clients about pregnancy danger signs than the highest-skilled cadre of registered/enrolled nurses. Provider performance is considered to be directly proportional to skills obtained after training and motivation, which includes being appreciated by the supervisors, as well as having a stable income and the opportunity for increased training, while is considered to be inversely proportional to barriers such as low salaries and poor working conditions [19,20]. Our results suggest that the poor performance shown by the registered/enrolled nurse cadre could be due to poor motivation and poor knowledge of the importance of counselling on danger signs. Such findings would support a qualitative study conducted in Northern Tanzania, which described several barriers to the provision of care and included poor supervision from the district level, delayed promotions, as well as lack of feedback on the cases referred to the higher level [21].

The auxiliary nurse cadre was the primary source of providers for 6 of the 14 dispensaries. This cadre has limited training in providing maternal care, but due to severe human resource for health shortage in the country, they are staffing a significant proportion of dispensaries and higher health facilities [21,22]. Studies have shown that using well-trained and skilled providers leads to better counselling and provision of maternal care [16]. Despite the poor performance of the highest skilled cadre we still believe that to improve counselling on pregnancy danger signs, as well as the quality of antenatal care in general, there is a clear need to increase trained personnel at all health facilities in the district. Nurse auxiliaries should be given priority for further training to higher cadres in order to increase this human resource base.

The interaction time between clients and providers was of short duration. The mean duration of client-provider interaction in the first visit was 17 minutes, while in the revisit was 10 minutes. It is unlikely that any meaningful information with respect to danger signs and other health issues could have been provided. Short time of interaction between clients and providers has been reported in other countries [14,18,23]. It is estimated that FANC takes no less than 46 minutes for the first visit, and 35 minutes for revisits. Counselling alone takes 15 minutes in both first visit and subsequent visit [15]. Although it can be argued about whether the recommended time is realistic within the context of these maternal health dispensaries - which are staffed by a Clinical Officer and a nurse auxiliary or an MCHA - providing adequate services to include preventive and curative care requires an effort to be made so as to ensure that providers allow enough time for the transfer of important information to all clients attending antenatal care.

Fewer than half of the pregnancy danger signs informed to the clients were recalled during the exit interview. This can partly be explained by the short duration of the client-provider interaction time, but also by the type of communication that we observed where the provider only doled out information and did not encourage clients to ask questions. Similar findings of one-directional communication have been reported in Nepal [14] and Zimbabwe [23]. Provision of information during health education could also take into consideration sociocultural aspects and could target the creation of awareness and behavioural change [24]. Such a communication strategy could be reinforced during the interaction in order to address client concerns and to enable the women to make informed decisions.

A limitation in this study was the presence of an observer during the client-provider interaction, which might have improved provider performance, as well as reduced client openness, in response to the fact that they are being observed. However, it has been demonstrated that the effect of the presence of an observer is short-lived, falling steadily to the usual situation before observation started [25]. Given the poor performance by the providers, the presence of the observer did not seem to affect their routine provision of antenatal care.

## Conclusion

In this study, we conclude that there is suboptimal quality of counselling during antenatal care on pregnancy danger signs. In order to equip pregnant women within the community to make timely decisions for seeking care when a complication arises, the situation needs to be improved. Supportive supervision to all cadres of antenatal care providers should be made to enhance counselling of pregnancy danger signs. Nurse auxiliaries should be encouraged and given chance for further training and upgrading to improve their performance and increase human resource for health available in rural areas, where multiple types of care cadres are likely to be more limited.

## Competing interests

The authors declare that they have no competing interests.

## Authors' contributions

ABP, AC, DPU, GL, LN and ED devised the study design and contributed to data interpretation. ABP and DP contributed to data collection. ABP performed statistical analysis and drafted the first manuscript. LN helped with statistical analysis. All authors read, commented on and approved the final manuscript.

## Pre-publication history

The pre-publication history for this paper can be accessed here:

http://www.biomedcentral.com/1471-2393/10/35/prepub
